# Down‐regulation of Polo‐like kinase 4 (PLK4) induces G1 arrest via activation of the p38/p53/p21 signaling pathway in bladder cancer

**DOI:** 10.1002/2211-5463.13262

**Published:** 2021-08-22

**Authors:** Ziyi Yang, Haiyan Sun, Wenlong Ma, Kai Wu, Guoyu Peng, Tong Ou, Song Wu

**Affiliations:** ^1^ Shenzhen University Health Science Center China; ^2^ Institute of Urology The Third Affiliated Hospital of Shenzhen University (Luohu Hospital Group) China

**Keywords:** bladder cancer, G1 phase arrest, p38/p53/p21 pathway, polo‐like kinase 4

## Abstract

Polo‐like kinase 4 (PLK4) has been reported to contribute to tumor growth, invasion, and metastasis. However, the role of PLK4 in human bladder cancer (BC) remains unclear. Here, we demonstrate the regulatory function of PLK4 in human BC progression. PLK4 is overexpressed in BC cell lines and tissues, and its overexpression correlated with poor prognosis. Our transcriptome analysis combined with subsequent functional assays indicated that PLK4 inhibition can suppress BC cell growth and induce cell cycle arrest at G1 phase via activation of the p38/p53/p21 pathway *in vitro* and *in vivo*. Overall, our data suggest that PLK4 is a critical regulator of BC cell proliferation, and thus, it may have potential as a novel molecular target for BC treatment.

## Graphical abstract

We demonstrate that Polo‐like kinase 4 (PLK4) is overexpressed in human bladder cancer (BC) cell lines and tissues, and its overexpression correlates with poor prognosis. PLK4 inhibition suppresses BC cell growth and induces G1 phase arrest via activating the p38/p53/p21 pathway *in vitro* and *in vivo*. The data suggest that PLK4 might serve as a novel molecular target for BC treatment.

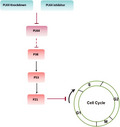

**Abbreviations**
BC: bladder cancer
CCK‐8: Cell Counting Kit‐8
GO: Gene Ontology
KEGG: Kyoto Encyclopedia of Genes and Genomes
MAPK: mitogen‐activated protein kinase
MIBC: muscle invasive bladder cancer
NMIBC: nonmuscle‐invasive bladder cancer
PBD: Polo‐Box domain
PLK4: Polo‐like kinase 4
TEMED: *N*,*N*,*N*′,*N*′‐tetramethylethylenediamine

Bladder cancer (BC) is a heterogeneous and complex disease, ranking the ninth among the most common cancers in the world. Meanwhile, both the incidence and fatality rates have been increasing year by year recently [1]. The majority of BCs belong to urothelial carcinoma and can be divided into non‐muscular invasive BC (NMIBC) and muscular invasive BC (MIBC). NMIBC accounts for 70–80% of newly diagnosed cases, but 10–20% of them may progress to MIBC, and the prognosis and the quality of life are much worse [2, 3]. However, the mechanism underlying the progression of BC remains elusive. Therefore, it is very urgent to discover novel molecular targets that may facilitate effective diagnosis and treatment.

Polo‐like kinase 4 (PLK4) is a cyclically dependent serine/threonine kinase containing only one Polo box at the C terminus, which makes it functionally different from other PLKs [4, 5, 6, 7]. As the upstream regulator of centriole replication, PLK4 plays an important role in mitosis regulation and DNA damage response [8, 9]. PLK4 dysregulation results in the aberrant centrosome numbers, thereby promoting mitotic catastrophe and tumorigenesis [10]. Previous studies reported that PLK4 was overexpressed in various tumors, including breast cancer [11, 12], lung cancer [13], neuroblastoma [14], colorectal cancer [15], malignant rhabdoid tumors [16], and prostate cancer [17]. However, the functions of PLK4 reveal different according to the types of carcinoma. For example, increased PLK4 expression promoted invasion and metastasis in breast cancer and neuroblastoma, while PLK4 expression declined during hepatocarcinogenesis, which makes it presumably being a tumor suppressor gene [18]. In addition, inhibition of PLK4 in genomic or pharmacologic could suppress tumor growth or increase sensitivity to radiation [19] and chemotherapy [12, 20, 21]. However, the biological function and mechanism of PLK4 in BC still remain unclear.

Because of the key roles in tumor growth and development, PLK4 is considered to be one of the most potential tumor therapeutic targets. Mak and his coworkers successfully screened and identified the most effective chemical molecule, CFI‐400945, as a potent and orally active antitumor agent for PLK4 inhibition. Treatment with CFI‐400945 *in vitro* induced aberrant centriole duplication and abnormal mitosis, further resulting in cell death or cell cycle arrest [22]. The potent antitumor effects of CFI‐400945 have been observed in multiple tumors, such as pancreatic cancer [23], Ewing's sarcoma [24], and lung cancer [13]. At present, clinical trials have been launched to evaluate the therapeutic potential in patients with advanced solid tumors and metastatic triple‐negative breast cancer [25, 26]. These encouraging findings implied potential application of CFI‐400945 in treating BC.

In this study, we investigated the expression patterns of PLK4 and its prognostic significance in BC for the first time. PLK4 knockdown assays combined with bioinformatics analyses demonstrated the involvement of PLK4 in regulating BC cell cycle arrest through the p38/p53/p21 pathway. Meanwhile, CFI‐400945 treatment exhibited consistent effects and molecular pathway with PLK4 down‐regulation, which might provide as a novel treatment strategy for BC patients.

## Materials and methods

### Data sources

The gene expression data of PLK4 were obtained from the GSE13507, Sanchez_Carbayo datasets, and GEPIA database (http://gepia.cancer‐pku.cn/). The prognostic information was downloaded from the GSE32894 and GSE13507 datasets. The expression of PLK4 mRNA in BC and normal tissues was verified using the Oncomine (https://www.oncomine.org) and The Gene Expression Omnibus (GEO) database (https://www.ncbi.nlm.nih.gov/geo). Immunohistochemistry (IHC) analysis was performed using a tissue microarray chip (Outdo Biotech Co., Ltd, Shanghai, China), which attached the clinicopathological and prognostic information of 68 BC patients.

### Cell culture

Human BC cell lines 5637 and MGHU3 (America Type Culture Collection, Manassas, VA, USA.), as well as normal human bladder epithelial cell line HCV‐29, were cultured in RPMI‐1640 medium supplemented with 10% fetal bovine serum (Gibco Laboratories, Gaithersburg, MD, USA) and 100 μg·mL^−1^ penicillin–streptomycin (Sigma‐Aldrich Co, St. Louis, MO, USA). Cells used in the animal experiments (6 × 10^6^) were cultured in RPMI‐1640 medium. All cells were grown in a humidified incubator under 37 °C and 5% CO_2_.

### RNA extraction, reverse transcription, and quantitative real‐time PCR

Total RNA of BC cells was extracted using Trizol reagent (TransGen Biotech, Beijing, China). The concentration of RNA was tested under 260/280 nm absorbance by NanoDrop ND‐1000 spectrophotometer (Thermo Scientific, Mass, USA), and the reverse transcription of 1 μg RNA was performed using the PrimerScript™ RT reagent Kit (Takara, Otsu, Japan). The quantitative real‐time PCR (qRT‐PCR) analysis was accomplished using the SYBR Premix Ex Taq II (Takara) on the 7500 Real‐Time PCR Detection System (Applied Biosystem, Foster City, CA, USA) platform. Relative quantitative expression of PLK4 and GAPDH was tested by 2^−ΔΔCT^ method analysis, and results were shown as fold change. The primer sequences used in this study for RT‐PCR were as follows: qPLK4‐F: 5′‐3′AGAAGCATTACATCTCCGTTGG, qPLK4‐R: 5′‐3′ACTCCTTTACAAGCTCCACAC; qGAPDH‐F: 5′‐3′CCATGGAGAAGGCTGG, qGAPDH‐R: 5′‐3′CAAAGTTGTCAGGATGACC.

### Transcriptome sequencing and analysis

Total RNA of shNC and shPLK4 BC cells (5637 and MGHU3) was isolated with Trizol reagent and purified using the RNeasy Plus Micro Kit (Qiagen, Shenzhen, China) according to the manufacturer's protocol. The RNA libraries were then sequenced using the Illumina HiSeq3000 at the BGI, Guangzhou, China. Fold change > 1.5 and *P*‐value < 0.05 were set as the threshold to screen differentially expressed genes (Table S1). To predict the possible functions and pathways of PLK4, the differentially expressed genes (shNC vs shPLK4 group) were entered into the Database for Annotation, Visualization and Integrated Discovery (DAVID, https://david.ncifcrf.gov/) for Gene Ontology (GO) and Kyoto Encyclopedia of Genes and Genomes (KEGG) analyses.

### Plasmid construction and transfection

Short hairpin (sh)RNAs against human PLK4 were subcloned into the AgeI/EcoRI sites of the pLKO.1 vector. The sequences of shPLK4‐1, shPLK4‐2, and the negative control were as follows: shRNA‐1: sense 5′CCGGCGTTGGTTGCTCACAGGTTAACTCGAGTTAACCTGTGAGCAACCAACGTTTTTG3′; shRNA‐2: sense 5′CCGGGACCTTATTCACCAGTTACTTCTCGAGAAGTAACTGGTGAATAAGGTCTTTTTG3′; shNC (negative control): sense 5′CCGGCCTAAGGTTAAGTCGCACCTCGCTCGAGCGCTTAGGTTTTTG3′.

For plasmid transfection, 293T cells (China Center for Type Culture Collection, Wuhan, China) were plated into 6‐cm dishes (2 × 10^6^ cells) and pLKO.1‐shRNA plasmids were cotransfected with virus packaging plasmids (pMD2.G; psPAX2) using TransIntro™ EL transfection reagent (TransGen Biotech). Then, cells were incubated for 48–72 h in the incubator. Collected lentiviral supernatants were filtered through a 0.45‐μm filter (Millipore, Burlington, MA, USA), and then, the lentivirus supernatants were added into 5637 and MGHU3 cells with the presence of polybrene (1 : 1000). After a total of 48‐h infection, cells were selected and exposed to puromycin (1 : 2000) with fresh complete medium for a week. Knockdown efficiency was verified by qRT‐PCR and western blotting.

### Western blotting

After corresponding treatments, cells were harvested and lysed using ice‐cold RIPA buffer (C1053, Applygen Technologies Inc., Beijing, China). Equal amounts of protein samples (20 μg per lane) were separated on 10% SDS/PAGE and then transferred onto the poly(vinylidene difluoride) membrane (Millipore). The membrane was then blocked with 5% milk followed by incubating with the primary antibody overnight at 4 °C and corresponding secondary antibody for 1 h at room temperature. Immunoreactive proteins were detected using the Immobilon Forte Western HRP substrate (Millipore, Cat. No. WBLUF0100) by BLT GelView 6000 Pro (Biolight Biotechnology Co., Ltd., Guangzhou, China). The primary antibody of this experiment includes phospho‐p38 mitogen‐activated protein kinase (MAPK; Thr180/Tyr182; Cell Signaling Technology, Danvers, MA, USA, 4511), p38 MAPK (Cell Signaling Technology, 8690), p53 (Cell Signaling Technology, 2524T), phospho‐p53 MAPK (Ser15; Cell Signaling Technology, 9286T), p21 (Cell Signaling Technology, 2947T), cyclin D1 (Cell Signaling Technology, 2978T), and GAPDH (Cell Signaling Technology, 5174T).

### Immunohistochemistry

After embedded with paraffin, tissues were deparaffinized and rehydrated with xylene and ethanol. Then, tissues were incubated in 3% H_2_O_2_ for 10 min to eliminate endogenous peroxidase activity. After blocked with 10% goat serum (diluted in PBS) for 10 min, tissues were incubated with primary antibody at the dilution of 1 : 50 and incubated at 4 °C overnight. Biotin‐labeled secondary antibody and horseradish phosphatase‐labeled streptavidin were then added and incubated for 30 min, followed by substrate reaction using DAB reagent. The reaction was stopped with tap water, and then, tissues were counterstained, dehydrated, and mounted. The primary antibody includes PLK4 (Proteintech, Rosemont, IL, USA, 12952‐1‐AP), phospho‐p53 MAPK (Ser15; Cell Signaling Technology, 9286T), phospho‐p38 MAPK (Thr180/Tyr182; Cell Signaling Technology, 4511), and p21(Cell Signaling Technology, 2947T).

### CCK‐8 assay

The impact of shPLK4 on the bladder cell proliferation was measured using Cell Counting Kit‐8 (CCK‐8; Beyotime, Haimen, China) assay according to the manufacturer's instructions. Briefly, cells were plated into 96‐well plates with 8000 cells per well. After 2 days, mixed CCK‐8 with medium at a dilution of 1 : 10 was added, and cell viability was analyzed for the next 6 days continuously. The absorbance of each group was measured by an enzyme‐labeled instrument (Multiskan GO 1510; Thermo Fisher Scientific, Waltham, MA, USA).

### Colony formation assay

To assess the colony‐forming ability after PLK4 knockdown, cells were seeded in 6‐well plates at a density of 2000 cells per well in complete medium for 2 weeks. The medium was replaced with fresh complete culture medium properly during the experiment. Colonies were fixed with ice‐cold methanol and stained with crystal violet (0.1%, Sigma‐Aldrich).

### EdU assay

In 5‐ethynyl‐2′‐deoxyuridine (EdU; RiboBio, Guangzhou, China) assay, BC cells with 1.5 × 10^5^ cells per well were cultured in confocal plates for 48 h and then exposed to 50 μm EdU for additional 4 h in the cell incubator. Cells were fixed with 4% formaldehyde for 15 min at room temperature, neutralized with 2 mg·mL^−1^ glycine treated, and then permeabilized using 0.5% Triton X‐100 for 20 min. Cells were reacted with 100 μL of 1×Apollo®reaction cocktail for 30 min at room temperature in the dark. Subsequently, cells were stained using Hoechst 33342 for 30 min and visualized under a fluorescent microscope (magnification 200×).

### Flow cytometry assay

Briefly, 2 × 10^6^ cells were harvested with trypsinization, washed, and fixed with 70% ethyl alcohol for 4 h under 4 °C, respectively. Then, cells were washed three times in 1 ml cold PBS. After dyed with Fxcycle PI/RNase Staining Solution kit (Invitrogen, Carlsbad, CA, USA) for 30 min at room temperature in the dark, the proportion of cells distributed in each phase was measured by BD FACSCalibur™ platform (BD, Becton, Dickinson and Company, Franklin Lakes, NJ, USA).

### Immunofluorescence assay

Cells (2 × 10^5^) were planted in confocal dishes and were added with CFI‐400945 for 24 h. The cells were washed with PBS and fixed with ice‐cold methanol for 2 min. Then, the cells were permeated and blocked with prepared buffer (PBS + 5% BSA + 0.3% Triton X‐100) for 40 min, followed by incubation with primary antibodies including α‐tubulin (1 : 1000; Proteintech, 66031‐1‐Ig) and γ‐tubulin (1 : 1000; Proteintech, 15176‐1‐AP) at 4 °C overnight. Cells were then washed and incubated with secondary antibodies including anti‐mouse IgG (H + L; Invitrogen, A21202) and anti‐rabbit IgG (H + L; Invitrogen, A31572) for 1 h at room temperature. DAPI was used to stain the nucleus. Stained cells were scored for multipolar anaphase cells using a confocal microscope (LSM800; Carl Zeiss, Oberkochen, Germany).

### Animal experiments

The research protocol for animal studies was approved and carried out in accordance with the Shenzhen University Institutional Animal Care & Use Committee. Female BALB/c‐nude mice (4–5 weeks) were purchased from Guangdong Provincial Experimental Animal Center. The mice were divided into two groups randomly. shNC or shPLK4‐2 cells of 5637 were mixed 1 : 1 with Matrigel (Corning Inc., Corning, NY, USA), respectively; then, cells (7 × 10^6^) were subcutaneously injected into the mice (*n* = 5). After 10 days, the tumor size was determined every 2 days. The length (*L*) and width (*W*) of tumor xenografts were measured to calculate the tumor volumes (*V* = *W* ^2^ × *L*/2), and the bodyweight was also measured. On day 28, the mice were euthanized for the subsequent experiments.

### Statistical analysis

graphpad prism software (GraphPad Software, version 8.0, San Diego, CA, USA) was used to evaluate the data. The results were presented as the mean ± standard deviation (SD) for at least three separate experiments. The significant differences between the control group and test group were analyzed by standard two‐tailed Student's *t*‐test. Kaplan–Meier was used to analyze OS. Comparisons were considered statistically significant when *P < 0.05, **P < 0.01, ***P < 0.001.

## Results

### PLK4 is overexpressed in BC patients and associated with poor prognosis

**Fig. 1:**
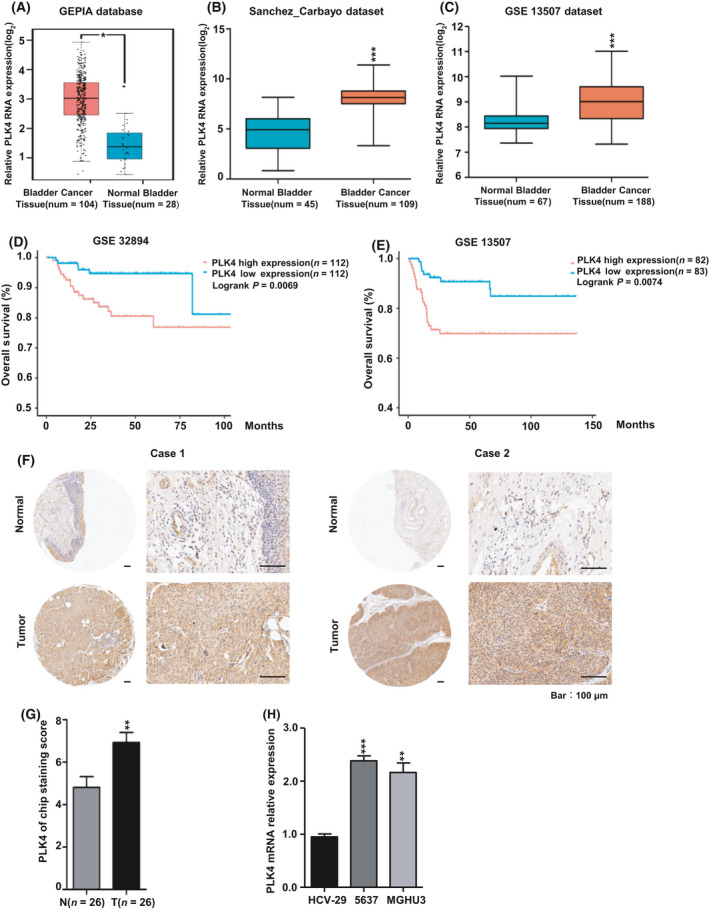
PLK4 is overexpressed in BC patients and associated with a poor prognosis. (A–C). PLK4 was overexpressed in BC tissues compared with normal bladder tissues according to the statistics from GEPIA (A), Oncomine (B), and GEO (C) databases. (D, E). Kaplan–Meier overall survival analysis for PLK4 expression level in BC patients using dataset GSE32894 (*n* = 224) and GSE13507 (*n* = 165) from the GEO database (*P* = 0.0069, *P* = 0.0074, respectively). (F). Representative IHC results of two pairs of normal and BC tissues (scale bar, 100 μm). (G). PLK4 staining score in BC and normal BC tissues is presented. (H). The mRNA level of PLK4 was significantly overexpressed in BC cell lines (5637, MGHU3) compared with the normal bladder epithelial cell line (HCV‐29). The *P* value was calculated using Student's *t*‐test. Results are shown as the mean ± SD. These results are representative of at least three independent replicates. *P < 0.05, **P < 0.01, ***P < 0.001.

We validated the differential expression of PLK4 in normal bladder tissue and BC in several databases. In Gene Expression Profiling Interactive Analysis (GEPIA) dataset (num(T) = 104; num(N) = 28) of TCGA & GTEx database, Sanchez_Carbayo dataset (num(T) = 109; num(N) = 45) of the Oncomine database, and GSE 13507 dataset (num(T) = 188; num(N) = 67) of the GEO database, high PLK4 mRNA expression was detected in BC samples compared with that in normal bladder tissues (*P < 0.05, ***P < 0.001, Fig. 1A–C), suggesting PLK4 as an oncogene of BC occurrence. Moreover, Kaplan‐Meier survival analyses in GEO database (GSE 32894, *n* = 224; GSE13507, *n* = 165) displayed that patients with high PLK4 expression had a significantly shorter overall survival (OS) than those with low PLK4 expression (*P* = 0.0069, *P* = 0.0074, respectively, Fig. 1D,E). Meanwhile, IHC analysis using a tissue microarray chip (Shanghai Outdo Biotech Co., Ltd.) further confirmed the high expression of PLK4 in BC patient samples (Fig. 1F); PLK4 staining score statistics were shown on the right (Fig. 1G, **P < 0.01). Besides, the same phenomenon was also reflected at the cellular level. As demonstrated in Fig. 1H, the mRNA expression level of PLK4 in BC cell lines, 5637 and MGHU3, was higher than that in normal bladder epithelial cell line, HCV‐29 (**P < 0.01, ***P < 0.001). These observations suggest that high level of PLK4 may act as a novel prognostic factor for BC patients.

### The RNA‐seq analysis of PLK4 knockdown in BC cell lines

**Fig. 2:**
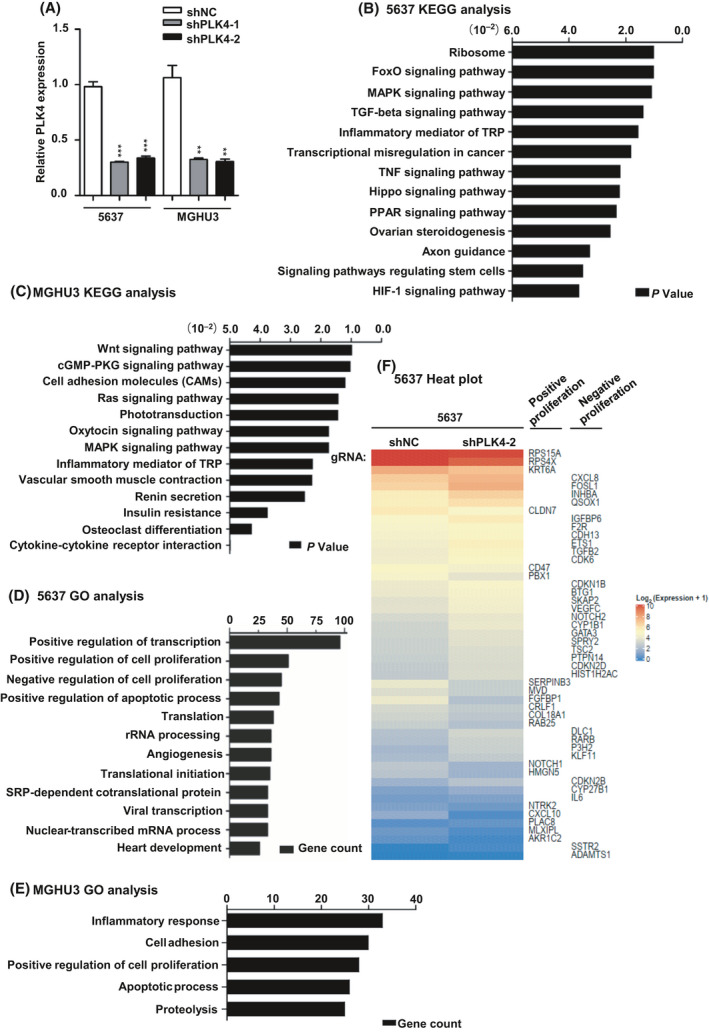
The RNA‐seq analysis of PLK4 knockdown in BC cell lines. (A). The mRNA expression level in PLK4 knockdown cell lines of 5637 and MGHU3 measured by qRT‐PCR. These results are representative of at least three independent replicates. (B, C) KEGG analysis of selected differentially expressed genes of 5637 (B) and MGHU3 (C) from RNA‐seq analysis. Bar plot shows the top enrichment score (*P*‐value) values for the significant enrichment pathways according to KEGG analysis of shNC and shPLK4 BC cells (5637 and MGHU3). Only pathways with *P* < 0.05 are shown. (D, E) The gene count of differentially expressed genes of 5637 and MGHU3 according to GO analysis. (F). Positive/negative regulation of cell proliferation in the knockdown cell pairs of 5637. The *P* value was calculated using Student's *t*‐test. Results are shown as the mean ± SD. **P < 0.01, ***P < 0.001.

To investigate the biological functions of PLK4 in BCs, we successfully established PLK4 knockdown cell lines of 5637 and MGHU3 (**P < 0.01, ***P < 0.001. Fig. 2A), followed by RNA‐seq analysis. We considered a 1.5‐fold change of expression and *P* < 0.05 as the standard from RNA‐seq data of 5637 to select 1245 differentially expressed genes (DEGs). Functional profiling analysis suggested that the affected genes were mainly enriched in biological processes including MAPK signaling pathway, FoxO signaling pathway, ribosome, and others (*P* < 0.05, Fig. 2B). Similarly, the MAPK pathway was also enriched in MGHU3 cell lines (Fig. 2C). Importantly, proliferation regulation gene was differentially expressed in the knockdown cell pairs according to GO function classification (Fig. 2D,E) and heatmap analysis (Fig. 2F), which indicated that PLK4 potentially contributes to BC tumorigenesis by regulating several relative signaling pathways and biological processes, especially the cell proliferation. It is known that the MAPK (mitogen‐activated protein kinase) family consists of three main subpathways: c‐Jun‐terminal kinase (JNK), extracellular signal‐regulated kinase (ERK‐1/2), and p38 mitogen‐activated protein kinase (p38 MAPK). These pathways have been confirmed to regulate various important physiological/pathological effects such as gene expression, cell division, cell survival, stress, and inflammatory response [27]. Considering the results of RNA‐seq analysis, we reason that PLK4 knockdown‐mediated inhibition of BC cell proliferation may associate with the MAPK signaling pathway.

### PLK4 knockdown reduces the proliferation of BC cells

**Fig. 3:**
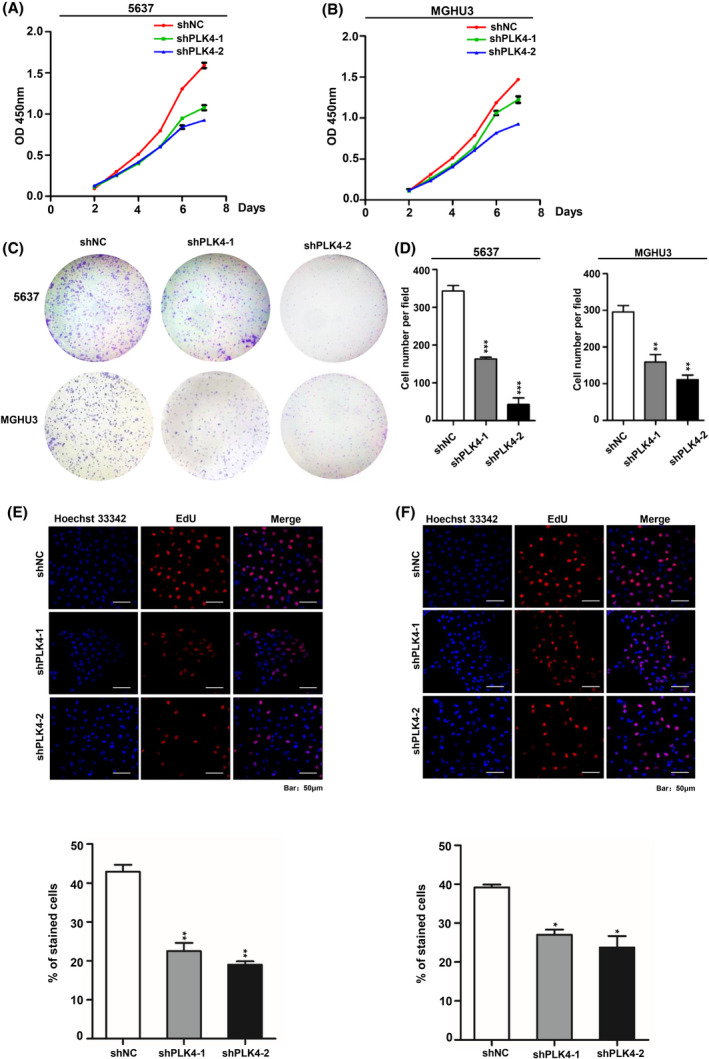
PLK4 knockdown reduces the proliferation of BC cells. A‐B). Effects of PLK4 knockdown on 5637 and MGHU3 cell proliferation, as evaluated by CCK‐8 assays. C‐D). Effects of PLK4 knockdown on 5637 and MGHU3 cells colony formation. Colonies that were visible and larger than 50 μm in diameter were counted. E‐F). EdU staining of control and PLK4 knockdown cells. The cellular DNA replication was inhibited in PLK4 knockdown cells (scale bar, 50 μm, blue: Hoechst 33342, red: the new generation cells, Magnification: 200×). The *P* value was calculated using Student's *t*‐test. Results are shown as the mean ± SD. These results are representative of at least three independent replicates. *P < 0.05, **P < 0.01, ***P < 0.001.

Based on the results of RNA‐seq analysis, several cell function experiments were conducted to verify the effect of PLK4 knockdown on BC cell proliferation. CCK‐8 assay showed that the proliferation capacity of PLK4 knockdown BC cells obviously decreased compared with the control group (Fig. 3A,B). The clonogenic assay revealed that clone size and the number of shPLK4‐1 and shPLK4‐2 BC cell lines were smaller compared with those of the control group (**P < 0.01, ***P < 0.001. Fig. 3C,D). In contrast, PLK4 knockdown displayed less effects on the proliferation of the human bladder epithelial cells (HCV‐29), suggesting that slow‐growing HCV‐29 cells were less sensitive to PLK4 inhibition (Fig. S1). Moreover, we performed EdU staining to further describe the proliferation inhibition more quantitatively. As shown in Fig. 3E,F (*P < 0.05, **P < 0.01, ***P < 0.001), cell proliferation ability is clearly displayed with a fluorescence microscope. Compared with the control group, the number of EdU‐positive cells displayed a significant decrease in shPLK4‐1‐ and shPLK4‐2‐treated BC cells.

### Inhibition of PLK4 induces cell cycle arrest at the G1 phase via p38/p53/p21 pathway

**Fig. 4:**
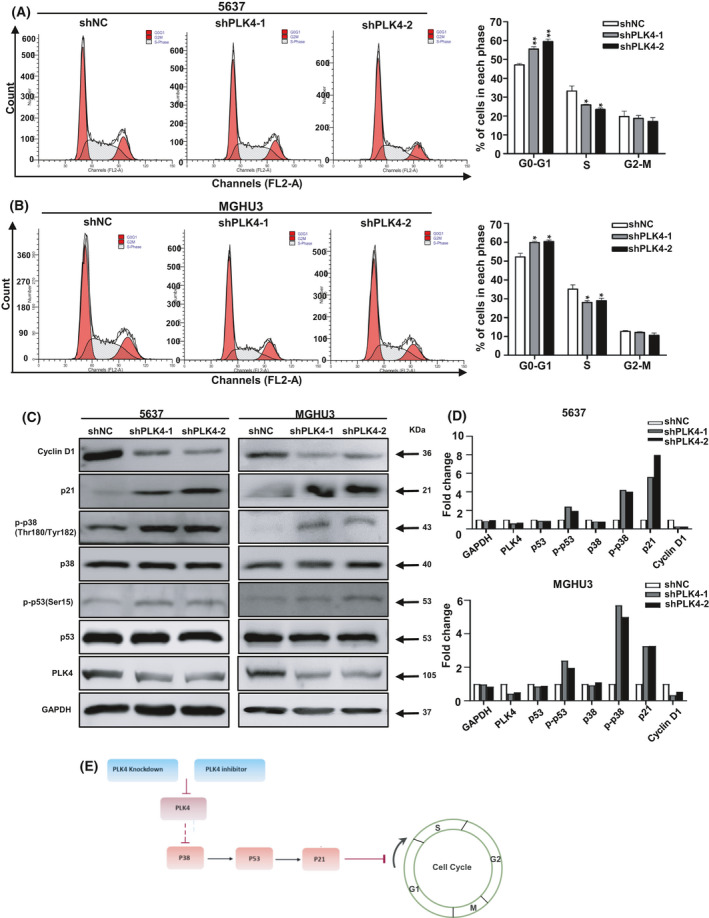
Inhibition of PLK4 expression induces cell cycle arrest at the G1 phase via p38/p53/p21 pathway. (A, B). Flow cytometry analysis to detect cell cycle distribution in PLK4 knockdown and control group of 5637 and MGHU3 cell lines. (C). Western blot assay to detect the expression of cell cycle arrest‐related proteins (p53, p‐p53, p38, p‐p38, p21, and cyclin D1) in PLK4 knockdown and control group of 5637 and MGHU3 cell lines. Data shown are representative images of three independent experiments. (D) Average fold change of each important protein with respect to corresponding control shNC. (E) A schematic demonstrating the possible mechanism of G1 arrest and proliferation inhibition induced by PLK4 down‐regulation. The *P* value was calculated using Student's *t*‐test. Results are shown as the mean ± SD. These results are representative of at least three independent replicates. *P < 0.05, **P < 0.01.

It has been reported that cell proliferation inhibition might be the result of cell cycle arrest. The flow cytometry analysis was conducted to investigate whether PLK4 knockdown inhibited BC cell proliferation through cell cycle regulation. As shown in Fig. 4A,B, compared with the control group, both of PLK4 knockdown groups displayed an increasing percentage of the G1 phase in the two BC cell lines. PLK4 is found mainly located in the centrosomes and closely related to the number and integrity of centrosomes. Previous studies found that loss of centrosome integrity could induce mitotic response involving p38 and p53 signaling protein. As a protein kinase, p38 can phosphorylate and activate p53 protein, further modulating the progression of the cell cycle [28]. Based on previous reports and our RNA‐seq analysis, western blot assay was performed to detect relative genes at the protein level. As shown in Fig. 4C,D, PLK4 knockdown had no influence on total p38 and p53 level, but obviously promoted the phosphorylation of them. We also analyzed cell cycle marker expression by western blot. In response to PLK4 knockdown, cyclinD1 expression decreased, which indicated G1 arrest. Moreover, p21 is an important cell cycle regulator that can arrest cell cycle progression at the G1/S phase. We found that p21 expression increased following PLK4 knockdown. The above evidence implied the important involvement of the p38/p53/p21 pathway in BC cell proliferation inhibition mediated by PLK4 suppression. The schematic demonstrating the possible mechanism of G1 arrest and proliferation inhibition induced by PLK4 down‐regulation is shown in Fig. 4E. We also showed the relevant original blots in Fig. S2.

### Targeted inhibition of PLK4 with CFI‐400945 affects BC cell proliferation and induces G1 arrest

**Fig. 5:**
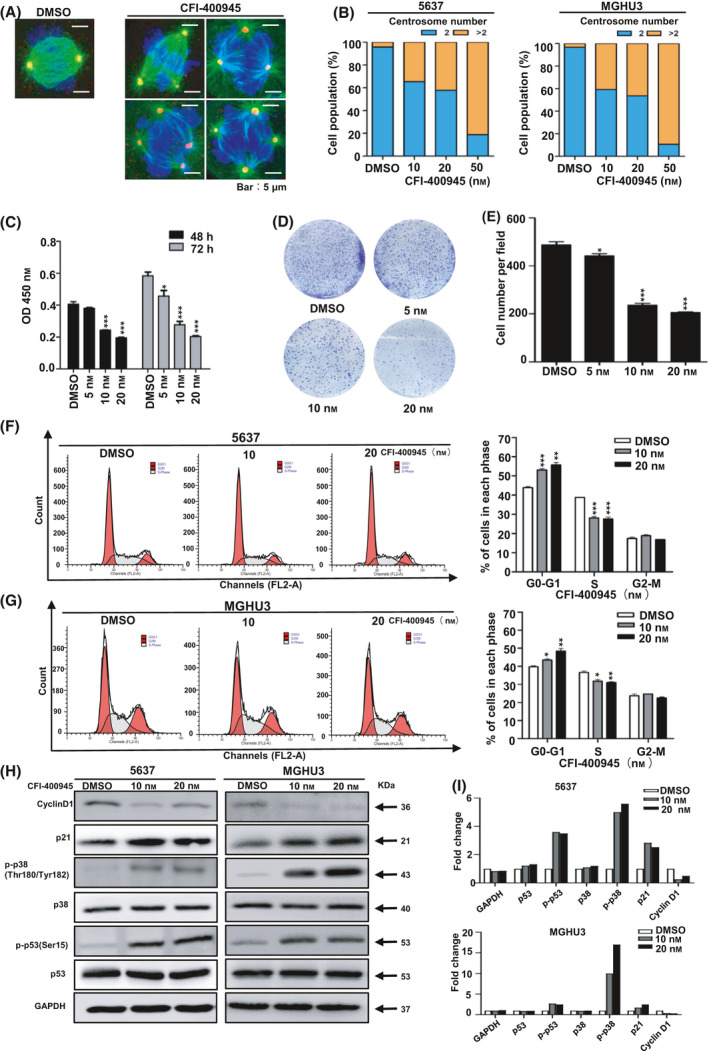
Targeted inhibition of PLK4 with CFI‐400945 affects BC cell proliferation and induces G1 arrest. (A). Representative images showing the cells with two or more centrosomes before and after CFI‐400945 treatment. (Scale bar, 5 μm, blue: DAPI, green: α‐tubulin staining, red: γ‐tubulin staining. Magnification: 63×). (B) Percentages of cell populations were displayed according to centrosome number. (C). CCK‐8 assay was used to examine the proliferation of 5637 cells treated with CFI‐400945 at different concentrations (5, 10, 20 nm; DMSO as the control) for 48 and 72 h. (D, E). Colony formation assays of 5637 cells treated with CFI‐400945 at different concentrations (5, 10, 20 nm; DMSO as the control). (F, G) Flow cytometry analysis to examine cell cycle distribution of 5637 and MGHU3 treated with CFI‐400945 (10, 20 nm; DMSO as the control). (H) Western blot assay to detect the expression of cell cycle arrest‐related proteins (p53, p‐p53, p38, p‐p38, p21, and cyclin D1) in 5637 and MGHU3 cells treated with CFI‐400945 (10, 20 nm; DMSO as the control). Data shown are representative images of three independent experiments. (I) Average fold change of each important protein with respect to corresponding DMSO group. The *P* value was calculated using Student's *t*‐test. Results are shown as the mean ± SD. These results are representative of at least three independent replicates. *P < 0.05, **P < 0.01, ***P < 0.001

The activity of PLK4 is closely related to the centrosomes [29]. Centrosome number and mitotic status were scored after CFI‐400945 or DMSO treatments by staining cells with γ‐tubulin, α‐tubulin, and DAPI. Representative images are shown in Fig. 5A. After 24‐h exposure, CFI‐400945 treatment caused an obvious increase in both cell populations with supernumerary centrosomes (*n* > 2) and multipolar spindles (Fig. 5B), indicating aberrant centrosome number and altered mitosis. The above results indicated that CFI‐400945 treatment inhibited the activity of PLK4. To investigate whether the inhibitor and PLK4 knockdown could produce a similar phenotype, we treated 5637 cells with different CFI‐400945 concentrations (DMSO, 5, 10, 20 nm). CCK‐8 assay showed that the inhibitory effect of CFI‐400945 was significantly enhanced as the concentration increased (Fig. 5C). Colony formation assay also confirmed the inhibitory effect of CFI‐400945 in BC cells (Fig 5D,E). Flow cytometric analysis demonstrated a cell cycle arrest at the G1 phase in CFI‐400945‐treated cells (Fig 5F,G). In addition, we observed the activation of p38 and p53 and the altered expression of downstream cell cycle regulators (p21 and cyclin D1) after CFI‐400945 treatment (Fig. 5H,I). The relevant original blots are shown in Fig. S2. In conclusion, the effects of CFI‐ 400945 are similar to PLK4 knockdown in terms of cell proliferation and G1 arrest in BC cells.

### Reduction of PLK4 suppresses BC growth *in vivo*

**Fig. 6:**
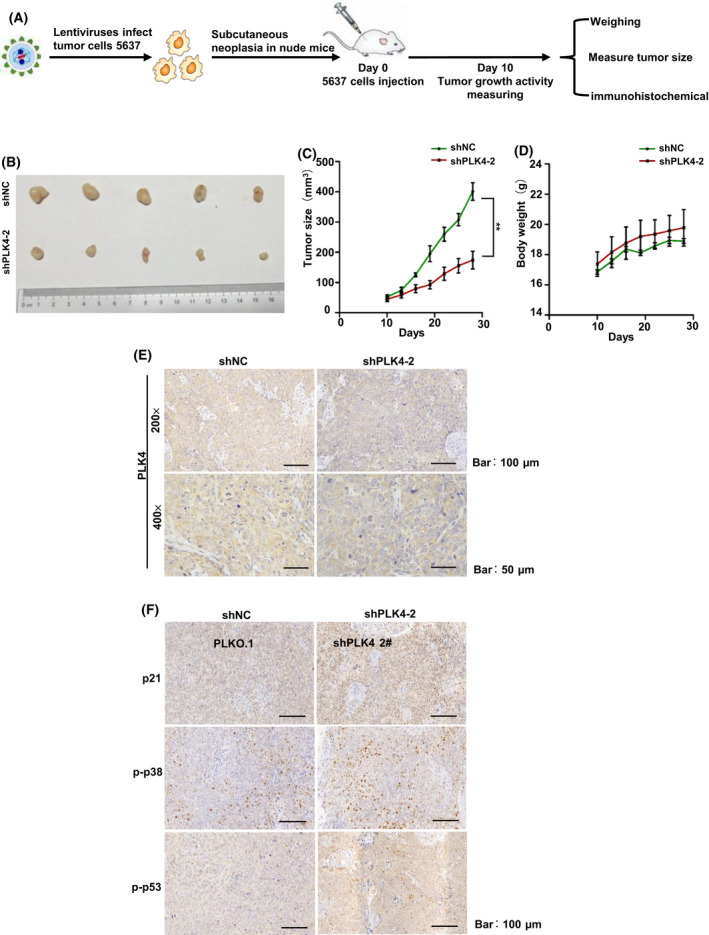
Reduction of PLK4 suppresses BC growth *in vivo*. (A). The mice tumor xenograft model of the shNC and PLK4 knockdown cell injection experiment (1 × 10^7^ cells per mouse, five mice/group, subcutaneous injection). (B) Bladder tumors from mice injected with shNC and shPLK4‐2 5637 cells. (C, D) Growth curves of xenograft tumors and bodyweight of the mice between the two groups. **P < 0.01. (E). IHC analysis of PLK4 expression in shNC and shPLK4‐2 xenograft tumors (200×, scale bar, 100 μm; 400×, scale bar, 50 μm). (F) IHC analysis of cell cycle arrest‐related proteins p‐p38, p‐p53, and p21 in shPLK4‐2 and control group (Scale bar, 100 μm). The *P* value was calculated using Student's *t*‐test. Results are shown as the mean ± SD. These results are representative of at least three independent replicates.

We then further explored the functions of PLK4 *in vivo* using the 5637 tumor xenografts assay. shNC and shPLK4‐2 cells (1 × 10^7^ cells per mouse) were subcutaneously inoculated into BALB/c nude mice, and all the operational procedure is shown in Fig. 6A. Compared with the control group, the tumor growth of the PLK4 knockdown group was significantly inhibited (Fig. 6B,C), while there was no significant difference in bodyweight between the two groups (Fig. 6D). Next, IHC staining was performed to testify the expression of PLK4. The results indicated that tumor tissues of the shPLK4‐2 group contained lower PLK4 expression than those of the shNC group (Fig. 6E). Moreover, the number of cells with p‐p38‐, p‐p53‐, and p21‐positive staining was higher in the shPLK4‐2 group than that in the control group (Fig. 6F), which was consistent with the results of PLK4 knockdown *in vitro*. In summary, these results indicated that the inhibition of PLK4 expression could suppress the growth of BC tumor *in vivo*.

## Discussion

Bladder cancer is a very complex disease and is caused by a variety of factors, including oncogenes and tumor microenvironment [30]. Previous reports have emphasized the importance of PLK4 in the development of various tumors [12, 14, 15]. However, few studies investigated its role in BC.

In our study, we firstly explored the expression of PLK4 using GEPIA online database, Oncomine database, and GEO database. We found that the mRNA level of PLK4 in BC was significantly higher than that in normal bladder tissue. Meanwhile, qRT‐PCR assay for BC cell lines and IHC staining for BC tissues about PLK4 expression showed consistent results with the datasets. More importantly, patients with a high PLK4 expression demonstrated a poorer prognosis according to the bioinformatic analyses of GSE32894 and GSE13507, further suggesting PLK4 as a potential prognostic factor in BC. PLK4 expression is closely related to centriole replication, and its aberrant expression can lead to an abnormal increase in the number of centrioles, causing centrosomes abnormalities, chromosome instability, and mitotic catastrophe [31]. Our experiments further confirmed that inhibiting PLK4 induced cell cycle arrest at the G1 phase, which was consistent with previous studies [32, 33].

Transcriptome analyses were performed to explore the potential regulatory mechanism of PLK4 in BC. KEGG analyses revealed the enrichment of the MAPK pathway in both 5637 and MGHU3 cell lines. The role of the MAPK pathway in tumorigenesis and immune disorders has been well recognized [34]. p38, one of the key members of the MAPK family, has been reported to be activated by the mitogen‐activated protein kinases via phosphorylating its T180/Y182 residue in response to environmental and mitogenic stresses [35, 36, 37]. Activated p38 stabilizes p53 through increasing p‐p53 expression [38, 39, 40]. In addition, p53‐dependent cell cycle arrest is mainly mediated by p21 [41]. Moreover, previous reports showed that disturbing centrosome‐related proteins could trigger cellular stress response involving in p38 and p53 signal proteins. In our western blot and IHC staining experiments, PLK4 inhibition promoted the phosphorylation and activation of p38 and p53, resulting in the up‐regulation of p21 and down‐regulation of cyclin D1, both of which are checkpoint markers of the G1 phase [42]. A recent report demonstrated that once centrosome disruption, USP28 would act together with 53BP1 to deubiquitinate and stabilize p53, leading to G1 phase arrest through regulating downstream protein p21. Therefore, another possible mechanism mediating cell cycle arrest in PLK4‐knockdown cells might be involved with USP28‐53BP1‐p53‐p21, which remains to be further explored in future. Next, we also confirmed the effect of PLK4 knockdown on BC tumor growth *in vivo*. The results revealed that PLK4 knockdown inhibited tumor growth, which was related to the activation of p38, p53, and p21.

Furthermore, we explored the effects of a selective PLK4 inhibitor (CFI‐400945) on BC cell lines (5637 and MGHU3). Consistent with the influence of PLK4 knockdown, CFI‐400945 treatment significantly inhibited the proliferation of BC cells and induced cell cycle arrest at the G1 phase. In addition, western blot analysis revealed that CFI‐400945 treatment could also remarkably activate the p38/p53 signaling pathway and induce p21 up‐regulation and cyclin D1 down‐regulation. Our observation indicated the potential clinical application of CFI‐400945 for BC therapy, although the antitumor activity and safety may need further investigation.

In summary, PLK4 overexpression is closely related to the poor prognosis of BC patients, and PLK4 suppression induces cell cycle arrest at G1 phase and inhibits BC cell proliferation via the p38/p53/p21 pathway.

## Conclusion

In this study, we confirmed that PLK4 has an association with promoting the development and progression of BC. PLK4 knockdown induces cell growth inhibition and G1 phase arrest of BC via regulating the p38/p53/p21 pathway, which indicated that PLK4 might be a critical molecular target for BC treatment.

## Author contributions

SW, TO, ZY, and HS conceived and designed the project. ZY, HS, and WM conducted experiments and performed data analysis. GP and KW contributed to the data acquisition and statistical analysis. ZY and HS wrote the original manuscript. TO and SW revised the manuscript. All authors read and approved the final manuscript.

## Conflict of interest

All authors declare no conflict of interest.

## Supporting information

**Fig. S1**. The effects of PLK4 knockdown on the proliferation of HCV‐29 cells. (A) Western blot assay to detect the expression of PLK4 in HCV‐29 cells. (B) Effects of PLK4 knockdown on HCV‐29 cell proliferation, as evaluated by CCK‐8 assays. (C). Effects of PLK4 knockdown on HCV‐29 cell colony formation. Colonies that were visible and larger than 50 μm in diameter were counted. The *P* value was calculated using Student's *t*‐test. Results are shown as the mean ± SD. These results are representative of at least three independent replicates. *P < 0.05.

**Fig. S2**. The original western blot membrane strips. (A) The original western blots in Fig. 4C. (B) The original western blots in Fig. 5H. (C). The original western blots in Fig. S1 (A).

**Table S1**. Differentially‐expressed genes.

## Data Availability

The gene expression data have been deposited in the GEO database under accession code GSE180958.
