# Supplementary figures and images for: Down‐regulation of Polo‐like kinase 4 (PLK4) induces G1 arrest via activation of the p38/p53/p21 signaling pathway in bladder cancer
Source: FEBS Open Bio. 2021 Aug 22;11(9):2631–46. doi: 10.1002/2211-5463.13262 (PMC8409300; doi:10.1002/2211-5463.13262)

Fig.S1

A

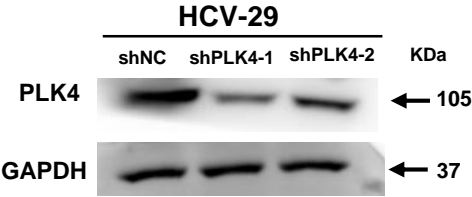

B

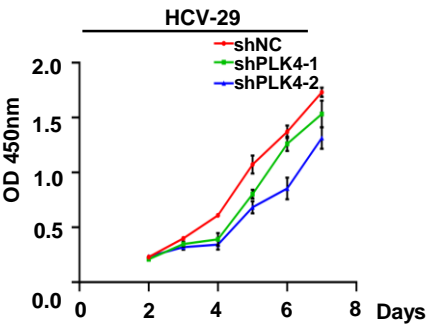

C

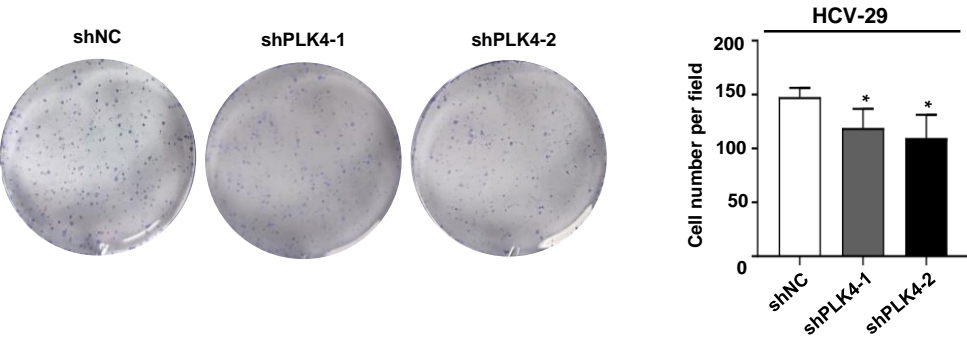

Supplement: Supplementary file 1 — Fig. S1. The effects of PLK4 knockdown on the proliferation of HCV‐29 cells. (A) Western blot assay to detect the expression of PLK4 in HCV‐29 cells. (B) Effects of PLK4 knockdown on HCV‐29 cell proliferation, as evaluated by CCK‐8 assays. (C). Effects of PLK4 knockdown on HCV‐29 cell colony formation. Colonies that were visible and larger than 50 μm in diameter were counted. The P value was calculated using Student's t‐test. Results are shown as the mean ± SD. These results are representative of at least three independent replicates. *P < 0.05. [file FEB4-11-2631-s002.pdf]

Fig.S2

Original western blot membrane strips

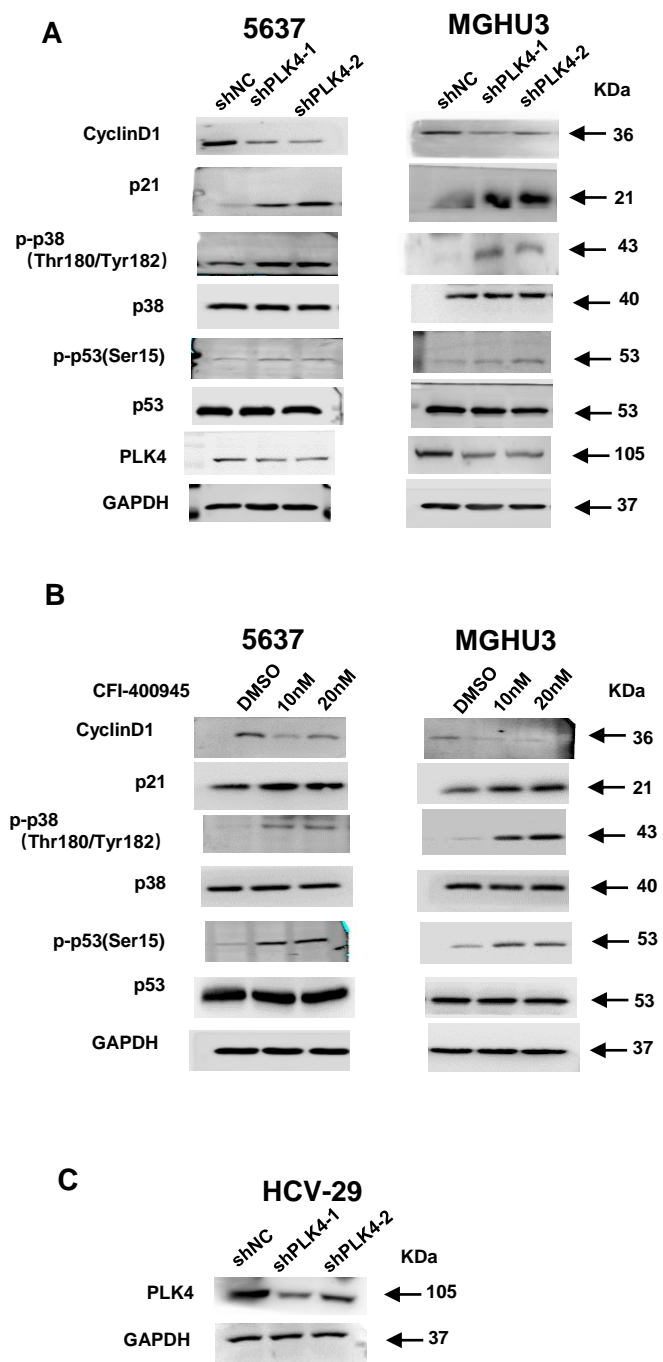

Supplement: Supplementary file 2 — Fig. S2. The original western blot membrane strips. (A) The original western blots in Fig. 4C. (B) The original western blots in Fig. 5H. (C). The original western blots in Fig. S1 (A). [file FEB4-11-2631-s003.pdf]
